# Age-associated changes in rich-club organisation in autistic and neurotypical human brains

**DOI:** 10.1038/srep16152

**Published:** 2015-11-05

**Authors:** Takamitsu Watanabe, Geraint Rees

**Affiliations:** 1 Institute of Cognitive Neuroscience, University College London, 17 Queen Square, London, WC1N 3A, United Kingdom; 2 Wellcome Trust Centre for Neuroimaging, University College London, 12 Queen Square, London WC1N 3BG, United Kingdom

## Abstract

Macroscopic structural networks in the human brain have a rich-club architecture comprising both highly inter-connected central regions and sparsely connected peripheral regions. Recent studies show that disruption of this functionally efficient organisation is associated with several psychiatric disorders. However, despite increasing attention to this network property, whether age-associated changes in rich-club organisation occur during human adolescence remains unclear. Here, analysing a publicly shared diffusion tensor imaging dataset, we found that, during adolescence, brains of typically developing (TD) individuals showed increases in rich-club organisation and inferred network functionality, whereas individuals with autism spectrum disorders (ASD) did not. These differences between TD and ASD groups were statistically significant for both structural and functional properties. Moreover, this typical age-related changes in rich-club organisation were characterised by progressive involvement of the right anterior insula. In contrast, in ASD individuals, did not show typical increases in grey matter volume, and this relative anatomical immaturity was correlated with the severity of ASD social symptoms. These results provide evidence that rich-club architecture is one of the bases of functionally efficient brain networks underpinning complex cognitive functions in adult human brains. Furthermore, our findings suggest that immature rich-club organisation might be associated with some neurodevelopmental disorders.

Large-scale structural networks in the human brain have so-called rich-club organisation, with densely inter-connected central regions but sparsely connected peripheral regions^1^,^2^,^3^,^4^. This architecture, which depends on long-distance connections, is anatomically costly^4^,^5^, but enables remote brain regions to communicate in an efficient and fast fashion, and thus is suitable for facilitation and integration of a wide range of complex information^4^,^6^,^7^,^8^. Recent work has suggested that this topological property could be altered in individuals with schizophrenia^9^,^10^, pre-adolescent children with neurodevelopmental disorders^11^, adults with experiences of maltreatment during childhood^12^, and individuals with Huntington’s disease^13^. A recent study has reported significant differences in the rich-club property of binary anatomical brain networks between teenagers and adults^14^, and there are similar differences in resting-state functional networks between pre-adolescent children and adults^3^.

Despite its potential importance for normal brain function, the age-associated trajectory of rich-club organisation during adolescence remains unclear. During the period, whole brain white matter volume increases while grey matter volume in several brain areas gradually decreases^15^,^16^,^17^. Alongside such maturation of white matter, both structural properties and functional performance of brain networks also change in the late teens^14^,^18^,^19^,^20^. However, the effect of such anatomical changes during adolescence on the rich-club structure is not fully understood.

Here, reflecting the functional efficiency of rich-club topology^4^,^6^, we hypothesised that brain networks in typically developing humans increase their rich-club properties during adolescence, and consequently develop efficient information processing. In addition, based on several theories describing autism as impairment of central processing of complex information^21^,^22^,^23^, we also hypothesised that rich-club structure might not be enhanced in such a way in autistic brain networks. We tested these hypotheses by assessing age-related changes in rich-club structure in typically developing (TD) adolescent brains and comparing the changes with those seen in brain networks of individuals with autism spectrum disorder (ASD). We also examined whether such age-associated alteration in rich-club architecture affected hypothetical measures of network functionality such as global efficiency^10^,^19^,^20^,^24^ and synchronisation cost^25^ in the two groups. Furthermore, to assess the anatomical importance of the network architecture in typical brain changes during the adolescence, we also compared age-related alternations in grey matter volumes (GMV) of rich-club regions between ASD and TD individuals. These analyses were performed based on publicly shared datasets of whole-brain structural connections^20^,^26^ and anatomical MRI images^27^ of ASD and age-/sex-/IQ-matched TD individuals.

## Results

### Younger v older individuals

**Figure 1:**
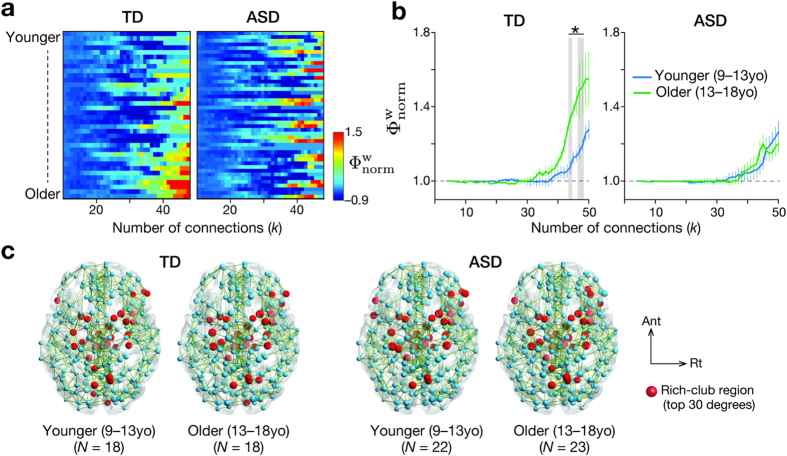
(**a**) Normalised rich-club coefficients for all individuals were sorted by age. TD, typically developing individuals; ASD, individuals with autism spectrum disorders. (**b**) In TD and ASD groups, the normalised rich-club coefficients were consistently >1 when the number of connections (degree, *k*) was large enough, suggesting rich-club organisation. In TD group, the older (13–18yo) had more apparent rich-club topology than the younger (9–13yo). In ASD group, no such difference was observed. Error bars, std. *P_FDR_ < 0.05 in *k* = 43, 47, 48. (**c**) Red circles represent rich-club regions, whereas blue circles show non-rich-club regions. The rich-club regions were defined as regions with top 30 degrees (i.e., the number of connections). For presentation purpose, the maps contain 3% most weighted connections (green lines). Ant, anterior; Rt, right.

Using datasets of undirected weighted anatomical connectivity matrices^20^,^26^ between 264 regions of interest (ROIs)^28^, we first calculated normalised weighted rich-club coefficients, 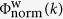, for each individual (Fig. 1a), and compared them between younger (9–13 yo) and older individuals (13–18 yo) (Fig. 1b,c, Supplementary Fig. 1, see Methods). In TD group, the rich-club coefficients at high degrees (*k* = 43, 47, 48) were significantly larger in the older individuals than in the younger individuals (*t*_34_ > 2.5, *P*_FDR_ < 0.05 in two-sample *t* tests). In contrast, such differences were not observed in the age-/sex-/IQ-matched ASD group at any degree (*t*_43_ < 1.2, *P* > 0.23).

This significant difference observed in TD group was robust against iteration of the age threshold dividing the group into younger and older individuals (*P*_FDR_ < 0.05 for iteration from 12 yo to 16 yo; 12 yo, *t*_34_ > 2.2 at *k* = 42, 43; 14 yo, *t*_34_ > 2.6 at *k* = 41–43, 46–48; 15 yo, *t*_34_ > 2.8 at *k* = 38–49; 16 yo, *t*_34_ > 3.7 at *k* = 42–49). Such difference was not seen in the ASD group for any of these age thresholds. There were no significant differences in 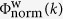 in the younger participants between TD and ASD groups at any degree (*P* > 0.6 in two-sample *t*-tests).

### Correlation between age and rich-club topology

**Figure 2:**
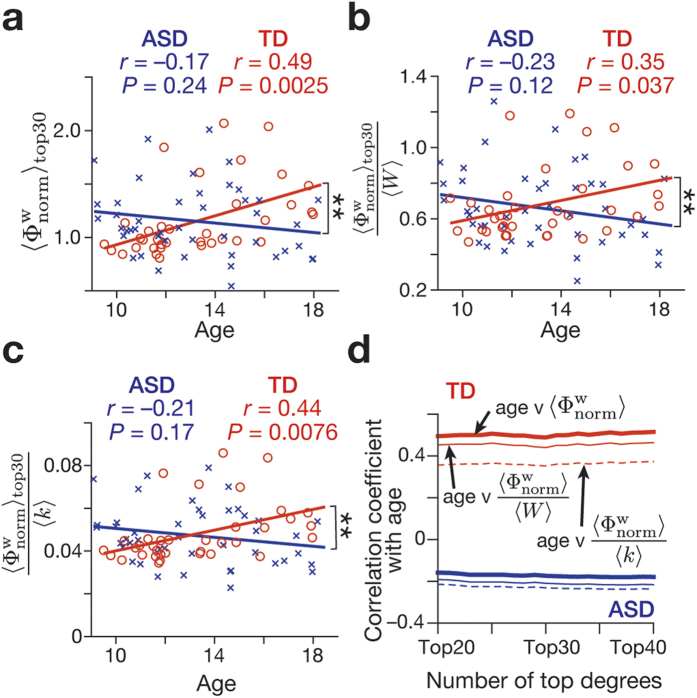
(**a**–**c**) In TD group (red circles), the average normalised rich-club coefficient among top 30 degrees was significantly correlated with age, whereas no such correlation was found in ASD group (blue crosses). There was a significant difference in the correlation between the two groups (*P* = 0.002) (panel **a**) This dissociable relationship with age was consistently observed when the rich-club coefficients were corrected by the average weights across region-pair (panel **b**) or the average number of connections (panel **c**) **P < 0.01. (**d**) The relationship between age and rich-club coefficient was consistently seen even when the number of top degrees was varied from 20 to 40 by one. Throughout the iteration, we observed significant difference in all types of the correlation coefficient between TD (red) and ASD (blue) groups (*P* < 0.05). Thick lines, correlations between age and average normalized rich-club coefficient; thin lines, correlations between age and weight-corrected rich-club coefficient; dashed lines, correlations between age and degree-corrected rich-club coefficient.

We then examined age-related changes in the rich-club property, which was quantified by averaging rich-club coefficients among the 30 largest degrees, 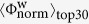 (see Methods). In the TD group, 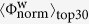 showed a significantly positive correlation with age (*r* = 0.49, *P* = 0.0025), whereas there was no such correlation in ASD group (*r* = –0.17, *P* = 0.24; Fig. 2a). The correlation observed in the TD group was significantly larger than that in the ASD group (*z* = 3.0, *P* = 0.002).

These correlations between age and rich-club topology were also evaluated using degree-controlled rich-club coefficient, 
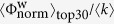
, and weight-controlled rich-club coefficient, 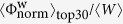, because age-related changes in white matter volumes were repeatedly reported^14^,^29^,^30^,^31^,^32^ (see also Methods and Supplementary Results). Consequently, we confirmed that the correlation patterns were qualitatively preserved in both types of rich-club coefficients (Fig. 2b,c).

In addition, this observation was robust even when we iterated the number of the top degrees from 20 to 40 (Fig. 2d). Moreover, this correlation pattern was essentially replicated when the rich-club property was quantified after excluding relatively noisy 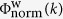 estimations seen at larger degrees (Supplementary Fig. 2). These results suggest that, in older TD individuals, high-degree rich-club regions tend to be more densely inter-connected, whilst brain networks of ASD groups are relatively stable in this point.

### Anatomical cost and network functionality

We then tested whether this age-associated enhancement of rich-club property brought an increase in anatomical cost and an improvement in functionality of brain networks.

**Figure 3:**
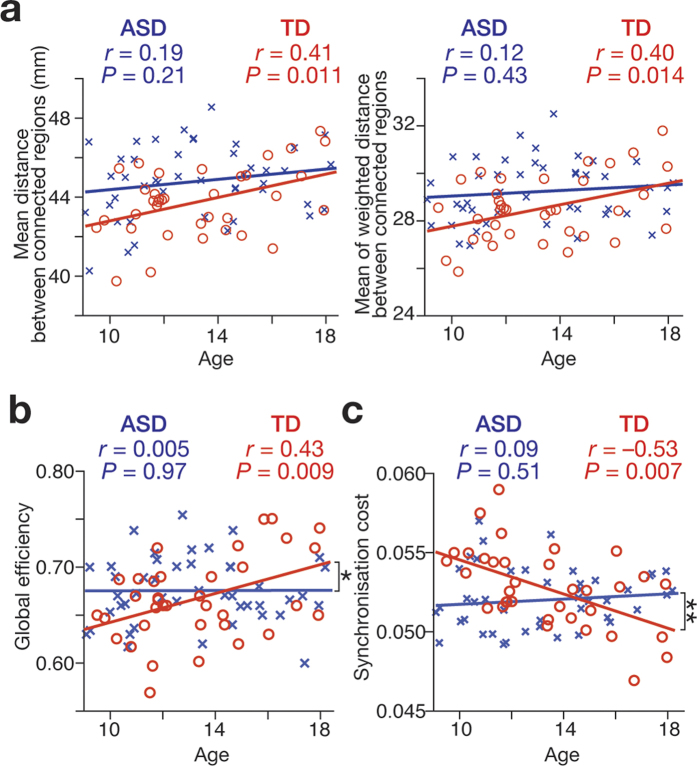
(**a**) In both simple anatomical distance (left panel) and weighted distance (right panel) between connected regions, correlations between age and the anatomical costs were significant in TD, whereas those were not in ASD individuals. (**b**) In TD group, global efficiency of the network was positively correlated with age, whereas no correlation was found in ASD group. There was a significant difference in the correlation between the two groups (*P* = 0.04). (**c**) Synchronisation cost was negatively correlated with age only in TD group. There was a significant difference in the correlation between TD and ASD groups (*P* = 0.005).

The anatomical cost was quantified as the average of unweighted anatomical distance between connected ROIs and the average of weighed distance between them (see Methods). In both indices, there was no significant difference in the age-related changes between TD and ASD groups (Fig. 3a). However, both types of the anatomical cost were significantly correlated with age in TD group (average unweighted distance: *r* = 0.41, *P *= 0.011, average weighted distance: *r* = 0.40, *P* = 0.014).

Two indices for the network functionality showed more dissociable patterns between TD and ASD groups: the global efficiency^9^,^10^,^19^,^20^,^24^ was positively correlated with age in TD groups but not in ASD groups (TD, *r* = 0.43, *P* = 0.009; ASD, *r* = 0.005, *P* = 0.97; TD v ASD, *z* = 2.0, *P* = 0.045; Fig. 3b); the synchronisation cost^25^ was negatively correlated with age only in TD (TD, *r* = –0.53, *P* = 0.007; ASD, *r* = 0.09, *P* = 0.51; TD v ASD: *z* = 2.9, *P* = 0.003; Fig. 3c).

Furthermore, these observations seen in the weighted networks were qualitatively replicated in analyses of binary unweighted networks (Supplementary Fig. 3, Supplementary Methods).

As a whole, these results suggest that in TD individuals, progressive enhancement of rich-club property is accompanied by changes of their brain networks into an anatomically costly but functionally efficient form, whereas such a transformation was not apparent in ASD individuals.

### Locational distribution of rich-club regions

**Figure 4:**
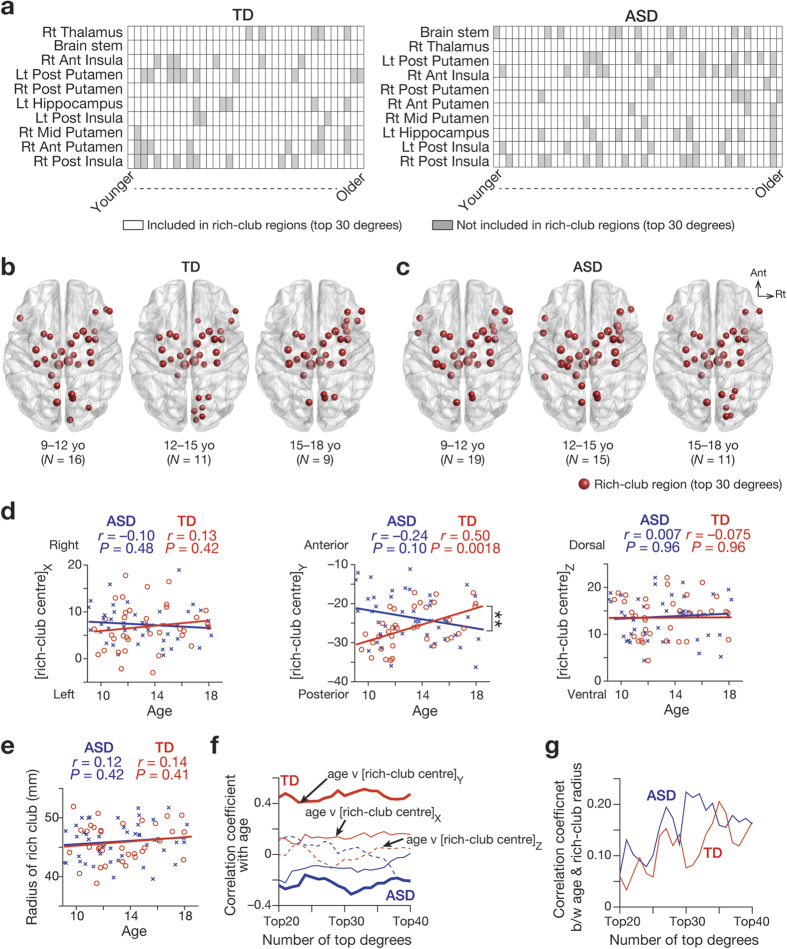
(**a**) Brain regions forming rich club. The matrices show patterns of brain regions’ appearing in rich club. Each column represents each individual: a white cell indicates that the region was included in rich-club regions with top 30 degrees; a grey cell indicates the opposite. The displayed regions are the 10 regions that most frequently appeared in rich club for each group. TD and ASD groups shared the 10 representative regions, though their appearance timing of these regions was different. Rt, right; Lt, left; Ant, anterior; Post, posterior; Mid, middle. (**b,c**) Distribution of rich-club regions. The red circles show the locations of rich-club regions with top 30 degrees of three age groups (Younger: 9 ≤ age < 12; Middle: 12 ≤ age < 15; Older: 15 ≤ age ≤ 18). The size of the circle represents the degree of each region. (**d,e**) Relationship between age and the rich-club centre and radius. In TD group, Y coordinate of the centre of rich club was positively correlated with age. The correlation was significantly different from that observed in ASD group (*P* = 0.0006). Such age-associated relationship was not found in X or Z coordinate (panel **d**), or the radius of rich club (panel **e**). (**f,g**) Robustness against the definition of rich-club regions. The correlations between age and locational distribution of rich club were robust against iterating the number of top degrees, which resulted in varying of the number of rich-club regions. Especially, the correlation between age and Y coordinate of the rich club centre was consistently observed in TD group, and its difference between TD and ASD groups were also coherently significant (*P* < 0.05; thick lines in panel **f**).

Next, we compared locational distributions of the rich-club regions with top 30 degrees between TD and ASD groups. The two groups shared the 10 regions that most frequently appeared in the rich club (Fig. 4a). Most of the 10 regions were found in the basal ganglia, and only insula was one of the cortical areas with a top 30 degree.

However, the temporal patterns of such ROIs appearing in the rich club were not the same between the two groups. Older TD individuals seemed to have more anterior regions in their rich clubs (Fig. 4b), whereas older ASD individuals appeared to have more posterior rich-club regions (Fig. 4c). This observation was supported by correlations between age and the locational information of the rich club (Fig. 4d,e): among X, Y, Z coordinates of the centre and the radius of rich club, only Y coordinate in TD groups was positively correlated with age, whereas that in ASD group showed a moderately negative correlation with age (TD, *r* = 0.50. *P* = 0.0018; ASD, *r* = –0.24, *P* = 0.10; TD v ASD: *z* = 3.4, *P* = 0.0006). These correlation patterns were consistently observed even when we iterated the number of top degrees and varied the range of rich-club regions (Fig. 4f,g).

**Figure 5:**
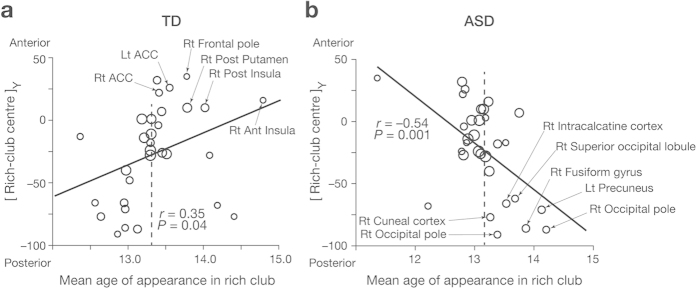
Rich-club regions were plotted based on their Y coordinates and the average age of appearance in rich club. If a given region appeared in the rich club irrelevantly to age, the average appearance age should be the same as the mean age of all individuals (13.3 yo in TD, 13.2 yo in ASD; a dashed line). In TD group (panel **a**), older individuals’ brains tend to include right anterior insula in their rich club; in ASD group (panel **b**), more posterior regions were likely to be included. The size of circle represents the appearance frequency in the rich club. Rt, right; Lt, left; ACC, anterior cingulate cortex.

Moreover, this differential antero-posterior change was confirmed by evaluating the association between Y coordinates of the rich-club regions and the average ages of individuals whose rich-club structure (i.e., ROIs with top 30 degrees) contained the given brain regions. In TD groups, more anterior brain regions tended to appear in rich club when individuals were older (*r* = 0.35, *P* = 0.04; Fig. 5a), whereas more posterior regions were likely to appear in rich club when ASD individuals were older (*r* = –0.43, *P* = 0.001; Fig. 5b). In particular, the right anterior insula (AI) seemed to largely contribute to this postero-anterior locational change of rich club in TD individuals.

### Anatomical examination of the specificity of right AI

This specific association of right AI with typical brain changes during adolescence was examined through evaluation of grey matter volume (GMV) of rich-club regions. This GMV analysis used a different anatomical MRI dataset^27^ because the original dataset for rich-club analysis did not contain the corresponding MRI data^20^,^26^ (see also Methods).

**Figure 6:**
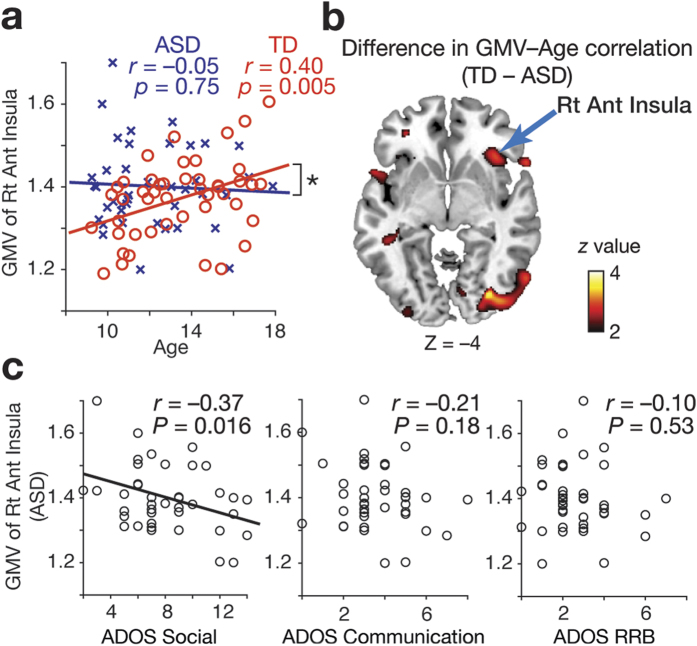
(**a**) GMV of right AI in TD individuals showed a significant positive correlation with age, whereas that in ASD individuals did not. *P < 0.05. (**b**) The difference in the GMV-age correlation between TD and ASD individuals was confirmed in a whole-brain analysis. (**c**) GMV of right AI in ASD individuals was correlated with severity of their deficits of reciprocal social interactions (left panel). The other major symptom scores did not have such correlations (middle and right panels). RRB: repetitive and restricted behaviours.

For this purpose, we calculated correlation coefficients between age and GMVs of the ten representative rich-club regions that were most frequently observed as ROIs with top 30 degrees in TD and ASD groups (i.e., the ten ROIs shown in Fig. 4a). Of the ten regions, only right AI showed a significant correlation with age in the TD group (*r* = 0.40, *P*_uncorrected_ = 0.00, *P*_FDR_ < 0.05; Fig. 6a), whereas no ROI showed such a significant association in ASD group (*P*_FDR_ > 0.05; Table 1). In addition, this age-GMV correlation in right AI in TD group was significantly larger than that seen in ASD group (*z* = 2.1, *P* = 0.03), which was also confirmed in a voxel-wise whole-brain GMV analysis (peak: [36, 26, –4], *z* = 3.0, *P*_uncorrected_ < 0.001; Fig. 6b). These results suggest that, compared with the other rich-club regions, the anatomical maturation of right AI plays a certain specific role in typical brain changes during adolescence.

### Anatomical evaluation of roles of right AI

Finally, we examined functional roles of the right AI by calculating associations between its GMV and severity of three main symptoms of ASD (impaired social interaction, communicational deficit, and repetitive and restricted behaviours), which were quantified by the Autism Diagnostic Observation Schedule (ADOS)^33^. We found that the GMV of right AI in ASD individuals was specifically correlated with the severity of autistic social deficits (i.e., social score in ADOS; *r* = –0.37, *P*_uncorrected_ = 0.016, *P*_FDR_ < 0.05; the other scores, *P* > 0.18; Fig. 6c). This relationship was preserved even after the correlation was calculated under the control of age effects (age-controlled partial correlation between GMV and ADOS social score: *rho* = –0.37, *P* = 0.019). These observations support the importance of anatomical maturation of right AI in typical brain changes, and suggest that its relative immaturity (as measured in terms of local GMV) might underlie deficits of social interactions in ASD individuals.

## Discussion

Here we found that, in typically developing adolescents, structural brain networks show progressively enhanced rich-club properties and thus may attain an anatomically costly but more functionally efficient form. In contrast, in ASD, such progressive enhancement of structural network rich-club properties is not seen. Moreover, the age-related changes in rich-club organisation in typically developing individuals necessitated involvement of right AI, and anatomical immaturity of this brain region was correlated with the severity of autistic social symptoms. These findings add evidence for rich-club properties being an important index for evaluating the age-associated changes in human brain networks and for delineating neurodevelopmental disorders.

The current findings on the relationships between age and rich-club organisation were based on the network datasets in which no fibres were excluded. Such an approach to exploit all the available data was adopted in previous studies^2^,^10^,^19^, but may leave some room for spurious fibres to still be included. In reality, all the main correlation patterns were qualitatively reproduced even after we excluded thin connections with fewer than five fibres (Supplementary Fig. 4). In particular, the difference between the younger and older participants in the TD group was qualitatively replicated in calculations based on group-averaged network matrices that more strictly excluded spurious fibres (Supplementary Fig. 5, Supplementary Methods)^1^. Furthermore, the correlation between age and the average rich-club coefficients was robust against the correction by the weight and degree (TD, *r* > 0.36, *P* < 0.03; ASD, –0.09 < *r* < –0.07, *P* > 0.64; TD v ASD, *z* = 2.0, *P* = 0.045). Given this robustness against exclusion of thin fibres, the typical age-related network changes in the brains might be attributed not to the absolute number of axon per se, but to the relative weight values assigned to white matter pathways that are supposed to depend on their microstructural properties such as myelination^34^.

The current observations about the age-related changes in rich-club coefficients are consistent with a recent report focusing on unweighted rich-club coefficients^14^. In contrast, another study reported no significant differences in rich-club properties of structural brain networks between TD children and neurotypical adults^3^. Methodologically, differences in scanning protocols might be relevant to this inconsistent observation: whereas the current main datasets are based on diffusion tensor imaging (DTI) data with 32 directions of angular resolution^26^, the previous study employed datasets with higher resolution (72 directions)^3^. Other factors underlying this inconsistency may be differences in the ROI definitions and in the number and characteristics of the participants. ROIs in the previous study^3^ consisted of 219 cortical regions that were determined by an algorithm called Connectome Mapper^34^, whereas the current 264 ROIs were based on a different previous study^28^ and additionally included 20 subcortical regions. Regarding the participants, the previous work studied a mixture of 29 healthy males and females, whereas the current findings about neurotypical individuals were based on data from 36 TD males. Although the current main findings were essentially replicated in modified brain networks excluding subcortical regions (Supplementary Fig. 6), these differences in ROI definition and sample property might result in the inconsistency between the previous and current studies.

Such methodological variations in determining brain networks may also underlie difference in locational distribution of rich-club regions between different studies. In fact, the current study found the ten regions in Table 1 as constituents of rich club, but not all the ten regions were reported as rich-club regions in previous studies that adopted different manners to construct brain networks from the current one. A previous study whose brain networks had a less number of ROIs (*N* = 82) than the current study found that, differently from the current result, rich-club regions included precuneus, superior frontal cortex, and superior parietal cortex^1^. Another study using a different neuroimaging protocol (high angular resolution imaging, HARDI), which is supposed to enable better estimation of anisotropy and detection of more fibres than conventional DTI^35^, reported that anterior/posterior cingulate cortex and inferior temporal cortex constituted rich club^11^. Given these discrepancies across studies, it appears to be necessary to attend the possibility that methods to build brain networks may affect determination of rich-club regions in anatomical brain networks.

The current findings about the network functionality in TD groups are consistent with previous reports^19^,^20^. Although it was based on binarised networks, the original study analysing the current dataset found that only in TD groups, characteristic path length, which is inversely associated with global efficiency, was negatively correlated with age^20^. Another study also reported a strong correlation between age and global efficiency that was measured in weighted brain networks of neurotypical individuals^19^. In addition, the current observations about the difference in the network functionality between TD and ASD groups provide further evidence for the importance of the index for characterisation of brain networks in psychiatric disorders: recent studies have reported significant dissociation in the global efficiency between healthy participants and individuals with several prevalent psychiatric disorders including schizophrenia^9^ and ASD^21^,^22^. These results imply that such age-associated changes in functional efficiency might be impaired in various types of psychiatric disorders.

The current observations provide a new context to interpret the age-associated changes found in brains of ASD individuals. As well as resting-state functional connectivity measured by functional MRI^20^,^36^,^37^,^38^,^39^, anatomical connections in ASD brains show several characteristic modulation patterns during adolescence^20^,^38^,^40^: in early childhood, white matter volume of ASD individuals tends to be larger than that of TD individuals, but subsequently it does not increase so much as in TD individuals^31^. White matter density in ASD individuals also shows a similar pattern and does not increase progressively during their teens^31^,^32^. A recent study has implied that some pathological disorganisation in cortical laminar architectures might underlie such atypical growth of ASD brains^41^. Moreover, another recent work has shown atypical large rich-club coefficients in pre-adolescent ASD children^11^. Considering these previous studies, the current findings might suggest that in early teens, rich-club coefficients for autistic structural brain networks are slightly larger in ASD groups than in TD groups, whereas structural network properties in ASD individuals are not particularly enhanced during early teenage years due to insufficient increase in white matter volume in ASD individuals.

The findings concerning right AI reported here also add evidence for the notion that dysfunction of this region underlies the social core symptoms of ASD^42^. This brain area is associated with particular types of social cognition such as empathy processing^43^, emotional judgements^44^, and reciprocal behaviours^45^ in TD individuals, and its hypo-activity during socio-communicational tasks has been repeatedly reported in previous neuroimaging researches studying ASD individuals^46^,^47^. In addition, recent work has shown atypical decreases in functional connectivity between right AI and other ASD-related focal brain regions including precuneus and superior temporal sulcus^36^,^42^. The current observation about the relatively smaller grey matter volume of right AI and its correlation with autistic social deficits is consistent with these previous findings, and implies the importance of the region for ASD.

Age-associated GMV changes in other rich-club regions are also compatible with a line of previous studies on anatomical characteristics of autism. First, the variety of the age-GMV relationships across regions is consistent with a previous observation that such age-related GMV changes are highly dependent on each region^48^. In addition, the current study found moderately larger increases in GMVs of thalamus and brain stem in TD group than ASD group (Table 1), which is in harmony with previous studies that GMVs of these areas in controls were larger than in those in individuals with high-functioning autism^49^,^50^. Moreover, the relatively small increase in hippocampal GMV in autism seen in the current study is also consistent with a previous meta-analysis that reported an ASD-specific decrease in GMV of the region^51^.

A limitation of this study is that we restricted our analysis to males and whether similar findings would hold in females is not clear: in fact, the original main data for rich-club analysis had only seven TD females and five ASD females in a narrow window of age. Moreover, the present findings are based on cross-sectional comparison across different individuals, rather than longitudinal within-participant comparisons^52^. Longitudinal studies are necessary for direct examination of the relationship between rich-club properties and the development of the human brain. Finally, we should also note that we did not include potential effects of individual differences in pubertal developments. Specifically, because the publicly shared datasets that we used did not hold information about whether each participant was pre- or post- puberty at the scanning time point, we could not precisely compare the difference in brain network structure between pre- and post- pubertal stages. Future studies might need to consider this potentially influential factor in investigating development of young brains^53^.

In conclusion, the current study found that rich-club topology of human brain networks was progressively enhanced during adolescence to have a more efficient form for information processing, whereas such enhancement of rich-club properties was not seen in the brains of individuals with autism spectrum disorder. Moreover, this typical age-associated changes in brain networks appeared to be largely dependent on progressive involvement of right AI, and the anatomical immatureness of this region was predictive of autistic social symptoms. These findings add further evidence for importance of rich-club organisation for typical age-related alternations of the human brain, and suggest that an immature rich-club topology might underlie some neurodevelopmental disorders.

## Methods

### Study design and ethics

The current study consisted of a graph theoretical analysis about rich-club organisation and a subsequent GMV analysis, both of which were performed on the basis of publicly-shared datasets^20^,^26^,^27^. As for the rich-club analysis, the data collection and sharing were approved by the UCLA Institutional Review Board and were conducted in accordance with the local institutional guidelines^20^, and those for the GMV analysis were approved by the corresponding local Institutional Review Boards (here, the New York University and the NYU School of Medicine Institutional Review Boards), and were performed in accordance with the corresponding institutional regulations^27^. In both datasets, the data were fully anonymised before being publicly shared, and written informed consents about the data collection and sharing were obtained from all the participants^20^,^26^,^27^.

### Data for analysis of rich-club topology

For analysis of rich-club organisation, we analysed structural connectivity matrices that had been used in a previous study employing high-functioning ASD individuals and matched TD participants^19^ and were shared in UCLA Multimodal Connectivity Database^26^. These connectivity matrices were built from DTI recorded by 3T MRI (Siemens Trio) in 8-min scans (32 directions b = 1000; 6 scans b = 50; 3 scans b = 0; 2 × 2 × 2 mm^3^ voxels). The DTI images underwent preprocessing consisting of brain extraction, eddy current correction by FMRIB’s Diffusion Toolbox (FMRIB’s Software Library, FSL)^54^, and motion correction by MCFLIRT^55^. Based on this preprocessing, participants showing excessive head motion were excluded (two TD and seven ASD males), and the resultant mean relative head motion was limited to 0.41 ± 0.13 mm for TD participants and 0.42 ± 0.11 mm for ASD individuals (mean ± SD).

The original study^20^ then mapped 264 ROIs including 20 subcortical ROIs, whose centre coordinates were determined by a previous study^28^, onto the individual brain surfaces using FSL (12 degree-of-freedom, affine, and correlation ratio cost function) through registration to the high-resolution images (12 degree-of-freedom, affine, and mutual information cost function). Consequently, each ROI was defined as a set of voxels whose distance from each centre coordinate was ≤10 mm on the continuous brain surface. The distance threshold was selected to ensure that the ROIs included enough nearby white matter fibres; in fact, ~60.1% of the voxels in the ROIs were averagely classified to white matter. This selection of spatially remote ROIs supposedly reduced the possibility of detecting false fibres.

Based on this surface-based ROI mapping, deterministic tractography between the 264 ROIs was performed using Fibre Assignment by Continuous Tracking (FACT) algorithm^56^ in Diffusion Toolkit^20^. First, a diffusion tenor model was fitted to the images, and fractional anisotropy (FA) values were calculated for each voxel by DTIFIT in FSL. Second, fibres were determined by propagating fibres from each voxel with a maximum turn angle set at 50°, which supposedly increases the detectability of long connections between spatially remote ROIs^57^. The detected fibres were then smoothed with a spline filter, and fibres shorter than 5 mm were removed. Then, the number of the fibres connecting two different ROIs was counted for all the possible combinations of the 264 ROIs. Finally, using the fibre counts as weights, 264 × 264 weighted structural connectivity matrices were built for all the participants. The following analysis used these weighted, undirected networks.

To minimise effects of sex differences on results, we focused on males, and used DTI data from 36 TD males (9–18 yo) and 45 autistic males (9–18 yo). The diagnosis of high-functioning ASD was based on DSM-IV, and confirmed with ADOS^33^ and/or Autism Diagnostic Interview (ADI-R)^58^ (ADOS communication + social, 10.8 ± 3.6; ADIR, 47.4 ± 11.6; mean ± SD). TD and ASD individuals showed normal intelligence (IQ ≥ 79), and did not have significant difference in their ages (*P* > 0.8).

### Estimation of rich-club coefficients

Using the structural connectivity matrices, we calculated normalised rich-club coefficients of these networks in the same manner as in previous studies^1^,^2^,^3^,^59^. Firstly, un-normalised weighted rich-club coefficient, Φ^w^(k), was calculated for each degree (i.e., the number of connections, *k*) as 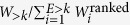, where *W*_>*k*_ is the sum of the weights of *E*_*>k*_ edges among *N*_*>k*_ regions with ≥*k* degrees, and 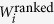 denotes the *i* th largest weight value among all weights. As a baseline, we also calculated the average 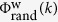 across 5000 random networks with equal degree and weight distribution^1^. Finally, the normalised weighted rich-club coefficient, 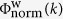, was estimated as the ratio of Φ^w^(*k*) to the average 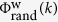 of random networks.

### Comparison of the rich-club organisation

We first compared 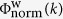 between the younger (age < 13) and older groups (13 ≤ age) for TD and ASD individuals. We set the threshold of age at 13 to balance sample size between the two age groups (TD: younger/older group, *N* = 18/18; ASD, younger/older group, *N* = 22/23). We then performed two-sample *t*-tests of 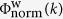 between the two age groups for each degree. These multiple comparisons across degrees were corrected by calculating false discovery rate (FDR).

Secondly, we examined associations between age and rich-club property. The rich-club architecture was quantified by averaging 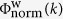 whose *k* was ranked in top 30 degrees, which is denoted by 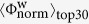. We also defined brain regions with these top 30 highest degrees as rich-club regions. Note that the number of the rich-club regions was not always equal to 30 because some regions might tie for, for example, the 30th-highest-degree position (rich-club regions in TD: 31.9 ± 2.4 regions; in ASD: 31.7 ± 1.6 regions; mean ± SD).

The degree threshold and definition of rich-club regions is comparable to that adopted in a previous study^11^, in which the threshold was determined as a degree from which 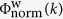 was continuously >1 and increased. If this approach is applied to the current estimation, the threshold will be approximately 35 (Fig. 1b), which is close to the current top30-based threshold (i.e., the 30th highest degree = 36.8 ± 2.7 in TD group and 37.6 ± 2.4 in ASD group, mean ± SD). Moreover, the proportion of the current rich-club regions (~10%) was similar to that in another previous study^1^. These similarities could justify the current degree threshold.

We next examined correlations between age and 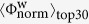 for each group. This correlation was also evaluated using degree-controlled rich-club coefficient, 
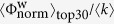
, and weight-controlled rich-club coefficient, 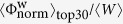, because age-related changes in white matter volumes have been reported in previous studies^14^,^29^,^30^,^31^ (see also Supplementary Results).

In addition, we also examined the robustness of these correlations against iterating the number of the top degrees from 20 to 40 by one, which consequently varied the fraction of rich-club region from ca. 8% to 15%.

Although a previous study suggests effects of an ASD-related gene on FA values in posterior regions^31^, we did not correct the rich-club coefficients by the fibre number within the posterior brain, because no significant correlations were found between age and the fibre number in the posterior area in TD or ASD group (|*r*| < 0.24, *P* > 0.11; posterior area: Y coordinate of ROI ≤ –15).

### Anatomical cost and network functionality

We then examined age-related changes in anatomical costs and functionality of the networks, because rich-club organisation is supposed to be anatomically costly due to its long-distance connections^4^,^5^ but to provide efficient interactions among brain regions^4^. To reduce the effects of age-related white matter changes^14^,^29^,^30^,^31^, we normalised the weights by dividing them by the average weight value in each individual.

The anatomical costs were quantified by averaging the Euclidian distances among connected brain regions^4^. We also evaluated another type of anatomical cost by calculating the average of the products of the distance and weights across connections (i.e., weighted distance).

Network functionality was measured by calculating global efficiency^9^,^10^,^19^,^20^,^24^ and synchronisation cost^25^. The global efficiency was estimated based on a previous study^24^, in which a weight value for each edge is regarded as a factor to enhance communications between the connected nodes. Technically, the distance between connected regions *i* and *j* was defined as 1/*W*_*ij*_, where *W*_*ij*_ is the connection weight between the two regions. We then estimated the shortest path length between all region pairs^24^, and calculated the global efficiency as (∑_*i*≠*j*_1/*l*_*ij*_)/*N*(N−1), where *N* represents the number of the regions and *l*_*ij*_ denotes the shortest path length between region *i* and *j*.

The synchronisation cost was calculated in essentially the same fashion as in a previous study^25^, in which the cost is supposed to represent a functional cost to keep synchronisation between coupled multiple oscillators. Technically, this synchronisation cost is defined in a complex network consisting of Kuramoto-type phase oscillators with different natural frequencies, which was determined by the following equation:


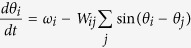


where *θ*_*i*_ and *ω*_*i*_ indicate the phase and natural frequency of the oscillator *i*, respectively. Note that *θ*_*i*_ is dependent on time *t*, whereas *ω*_*i*_ is a temporally constant. In such a classical Kuramoto model, even when the entire network reaches a frequency-synchronisation state at *t*→∞, the individual oscillators can have their own different phases 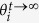. Because this phase difference can be regarded as a type of burden for each edge, the synchronisation cost, *S*_*ij*_, for an edge between nodes *i* and *j* was defined as 
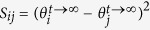
. We then calculated the average synchronisation costs across edges for each individual. In this calculation, the connection weights behave as a factor to enhance communications between connected regions, which keeps consistency with the above-mentioned estimation of global efficiency.

### Locational difference in rich-club regions

We then compared the locational distribution of rich-club regions between ASD and TD groups. First, in each group, we counted how many times each brain region appeared in the rich-club regions with top 30 degrees, and searched for the 10 most frequently appeared regions.

Second, we investigated age-related changes in the location of the rich club by calculating its centre and radius in each individual: the centre was defined as the average of the coordinates of the rich-club regions; the radius was calculated by averaging the distance between the centre and each rich-club region. We then assessed correlations between age and this rich-club locational information. The robustness of these correlations was confirmed by performing the same analysis with different definitions of the rich-club regions That is, we tested the findings by iterating the number of top degrees from 20 to 40 by one.

Finally, we quantified at what age each region was likely to appear in the “rich club” by averaging the ages of individuals whose rich clubs included the given region. To focus on brain regions that mainly contributed to rich-club organisation, this calculation was performed only for regions that appeared in rich clubs in >30% of individuals. We then plotted these regions based on their coordinates and the average of appearance ages, and attempted to find critical regions that are mainly responsible for the typical age-associated changes in rich-club organisation.

### Data for additional GMV analysis

Additionally, we examined the anatomical importance of the critical regions selected in the analysis above (here, anterior insula) by investigating associations between the GMV and age/symptoms. This additional analysis used a different dataset shared in Autism Brain Imaging Data Exchange (ABIDE)^27^, because the dataset for the main analysis does not include corresponding anatomical MRI images.

The T1-weighted MRI images consisted of 41 ASD and 46 age-/IQ-matched TD individuals that were recorded a 3T MRI (TR = 2.53s, TE = 3.25 ms, 1.3 × 1.0 × 1.3 mm, 128 slices) in NYU Langone Medical Centre in ABIDE. The diagnosis of ASD was performed based on DSM-IV-TR and was confirmed by ADOS. Their IQ was measured with WASI (ASD, IQ > 78; TD, IQ > 80). Here, to keep consistency with the rich-club analysis, we selected 9–18 year-old male participants from this dataset. Note that this second dataset could not be used for the analysis of rich-club coefficient because it did not include DTI data.

### Additional GMV analysis

We preprocessed the anatomical images in SPM8^60^: for each participant, the image was segmented into grey matter, white matter, and cerebrospinal fluids in the native space with the New Segment Toolbox^61^. The segmented grey matter images underwent alignment, warp to a template space, resampling down to 1.5 mm isotropic voxels, and registration to a participant-specific template using the DARTEL Toolbox^62^. The individual grey matter image was then normalized to MNI spaces and smoothed with a Gaussian kernel (FWHM = 8 mm). Finally, each image was normalized by the whole-brain GMV value.

Using these preprocessed data, we conducted a ROI analysis about the ten representative rich-club regions (i.e., the ten ROIs in Fig. 4a). For each group, we extracted GMVs of these ROIs (a 4-mm sphere), and then calculated their associations with age. The statistical significance of the obtained correlation coefficients was evaluated after they were corrected for multiple comparison by calculating FDR. Based on the obtained results, we then focused on right AI, and examined relationships between its GMV and ADOS scores.

To confirm locational specificity of this selection of right AI, we also performed voxel-based morphometry and compared the GMV-age associations between TD and ASD groups. Using the pre-processed whole-brain images, we performed regression analyses with age as an independent variable, and calculated a regression coefficient between GMV and age for each voxel, and then estimated difference in the regression coefficients between TD and ASD groups.

## Additional Information

**How to cite this article**: Watanabe, T. and Rees, G. Age-associated changes in rich-club organisation in autistic and neurotypical human brains. *Sci. Rep.* **5**, 16152; doi: 10.1038/srep16152 (2015).

## Supplementary Material

Supplementary Information

## Figures and Tables

**Table 1:** Correlations between age and GMV of rich-club ROIs.

Rich-club regions	TD	*P* value	ASD	*P* value	TD v ASD
	Correlation coefficient with age		Correlation coefficient with age		*P* value
Rt Ant Insula	0.4	0.00*	–0.05	0.75	0.03
Rt Post Insula	–0.06	0.69	0.28	0.07	0.12
Lt Post Insula	–0.1	0.5	0.3	0.05	0.065
Lt Hippocampus	0.22	0.14	0.09	0.57	0.55
Rt Ant Putamen	–0.03	0.84	0.14	0.37	0.44
Rt Mid Putamen	–0.19	0.2	0.001	0.99	0.39
Rt Post Putamen	–0.24	0.1	0.13	0.41	0.09
Lt Post Putamen	–0.21	0.16	0.03	0.85	0.27
Rt Thalamus	0.25	0.09	0.05	0.75	0.35
Brain Stem	0.21	0.16	0.14	0.37	0.75

*P_FDR_ < 0.05; Rt, right; Lt, left; Ant, anterior; Post, posterior.
