# Supplementary material for: Age-associated changes in rich-club organisation in autistic and neurotypical human brains
Source: Sci Rep. 2015 Nov 5;5:16152. doi: 10.1038/srep16152 (PMC4633620; doi:10.1038/srep16152)
Supplement: Supplementary Information [file srep16152-s1.pdf]

Supplementary Information
for "Age-associated changes in rich-club organisation
in autistic and neurotypical human brains."
by Watanabe and Rees.

Supplementary Figure 1

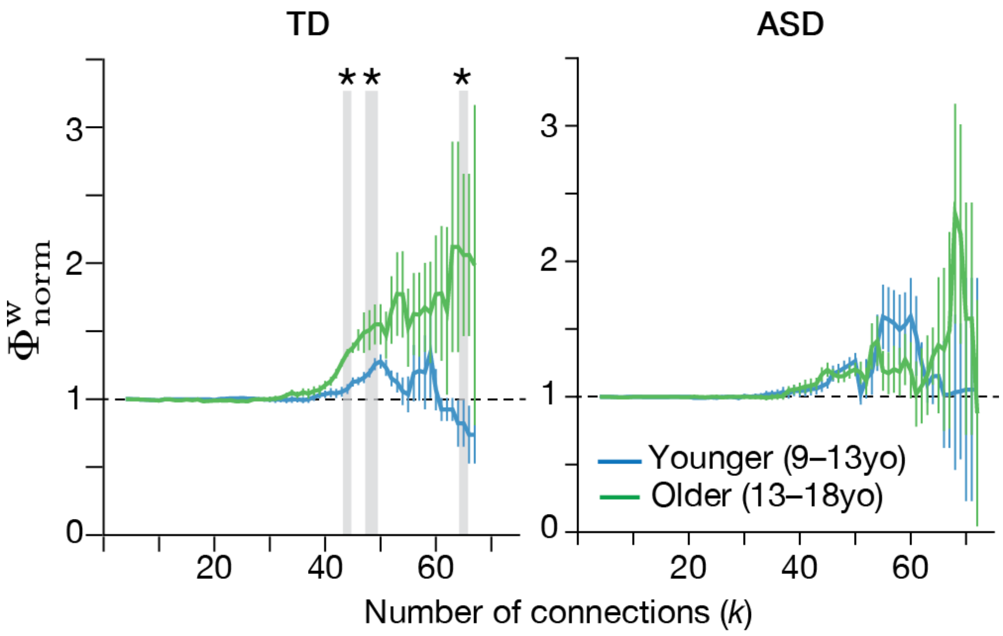

<u>Comparison of rich-club coefficients between young and old participants.</u>

The two panels show rich-club coefficients seen in an extended degree range. Qualitatively, in TD group, the coefficients seen in the older participants were larger than those seen in the younger participants

The estimation observed at >50 degrees were noisy because some participants' brain networks did not have enough number of ROIs to calculate rich-club coefficients at some high degrees. The panels did not present rich-club coefficients at some large degrees ($k > 69$ in TD, $k > 74$ in ASD), because it was impossible to estimate standard deviations at that range. The main figure (Figure 1b) focused on the reliable and meaningful part of this estimation ($k < 50$). *, $P_{\mathrm{FDR}} < 0.05$ in $k = 43, 47, 48, 65$.

Supplementary Figure 2

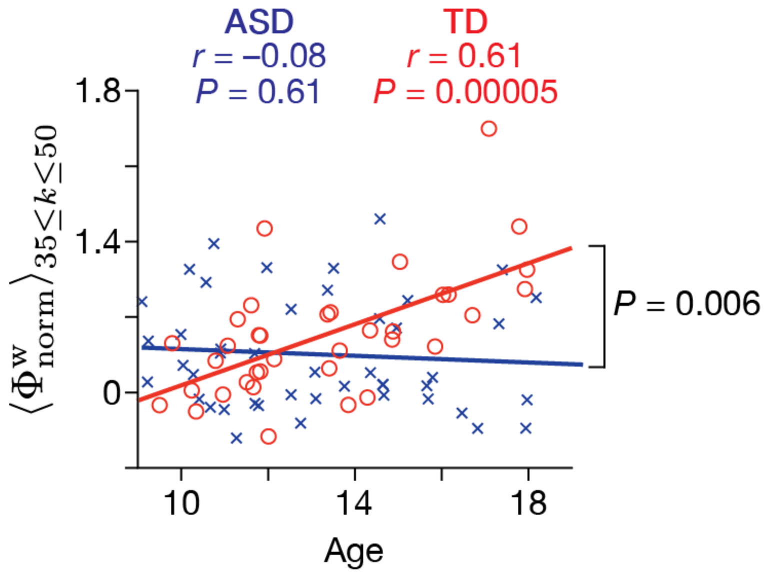

Relationship between age and rich-club coefficients ranging from $k = 35$ to $k = 50$.

The age-related changes in average rich-club coefficients seen in Figure 2 were also observed even when the coefficients were averaged across degrees ranging from 35 to 50. This degree range was determined based on a previous study[1]: first, Figure 1b shows that stable increases in the coefficients began at around $k = 35$ in all the TD/ASD and younger/older groups; in addition, Supplementary Figure 1 indicates that, when the degree was more than 50, the estimation of the coefficients appeared to be relatively noisy; therefore, according to the previous study, this analysis focused on the rich-club coefficients from $k = 35$ to $k = 50$.

Supplementary Figure 3

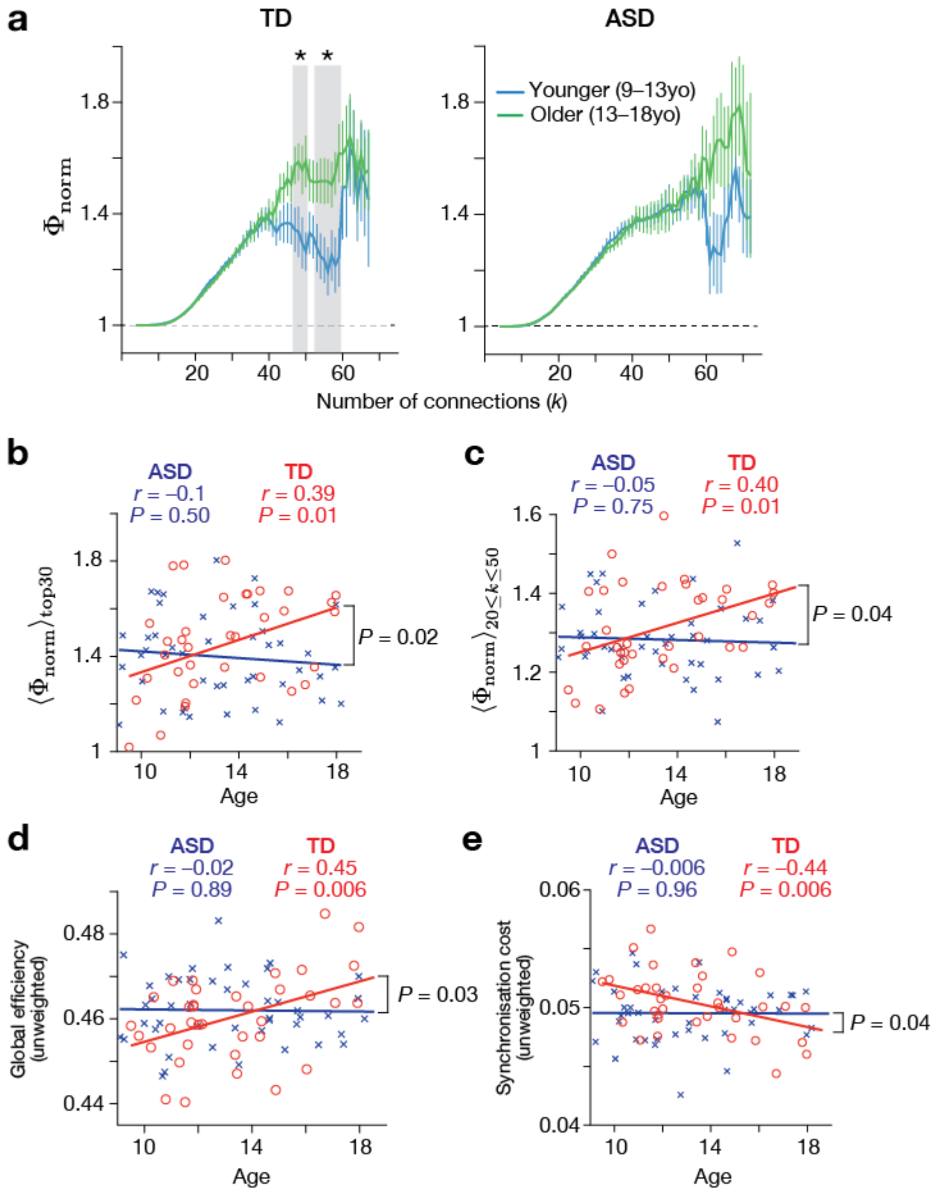

Results based on binary brain networks.

The analyses using binary unweighted brain networks showed qualitatively the same results as those seen in the analyses using weighted networks. **(a)** In TD group, the older group showed larger rich-club coefficients than the younger group. *, $P_{FDR} < 0.05$ in $k = 47–50, 53–59$. **(b)** The rich-club coefficients averaged across top 30 degrees were significantly correlated with age only in TD group. **(c)** The similar age-related increase was observed even when the coefficients were averaged across degrees from $k = 20$ to $k = 50$. The range of degree was determined in the same manner described in Supplementary Figure 2. **(d) (e)** Global efficiency and synchronisation cost also showed age-related changes only in TD groups.

Supplementary Figure 4

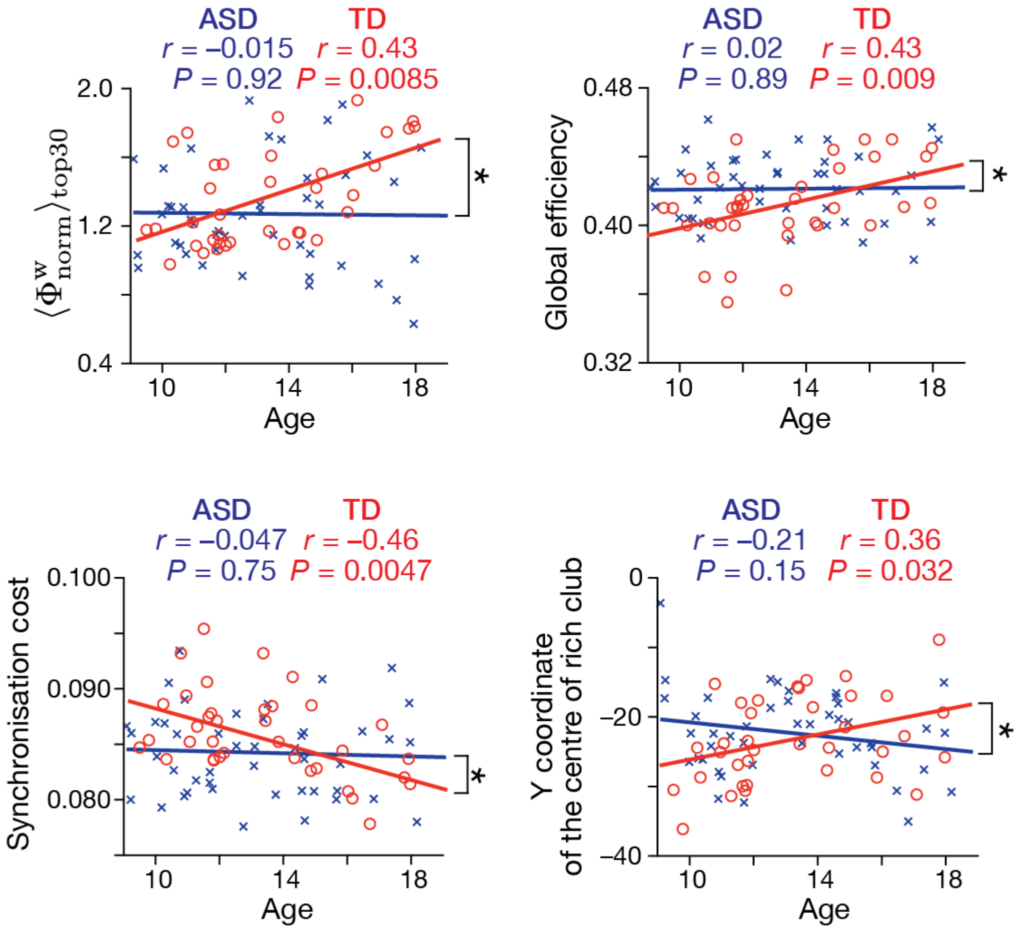

<u>Robustness against exclusion of thin connections.</u>

The main part of the current observations could be reproduced even when we excluded thin connections that had less than five fibres. *, $P \leq 0.05$.

Supplementary Figure 5

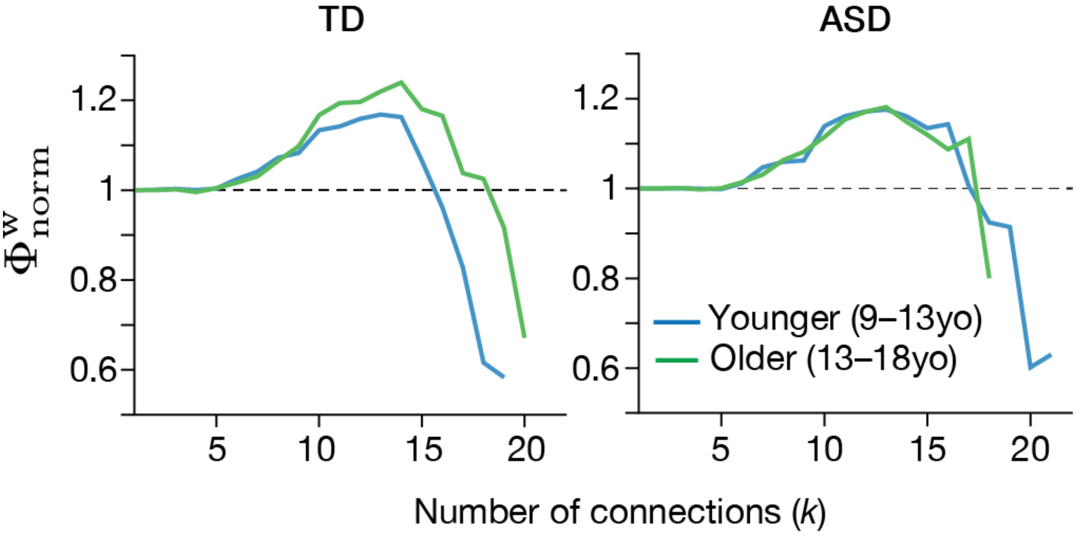

<u>Group-averaged rich-club coefficients.</u>

The panels show rich-club coefficients that were calculated from group-averaged weighted brain networks in the same manner as one described in a previous study[2] (Supplementary Methods). The older group shows larger values than the younger group only in TD group, which is comparable to the observations presented in Figure 1b.

Supplementary Figure 6

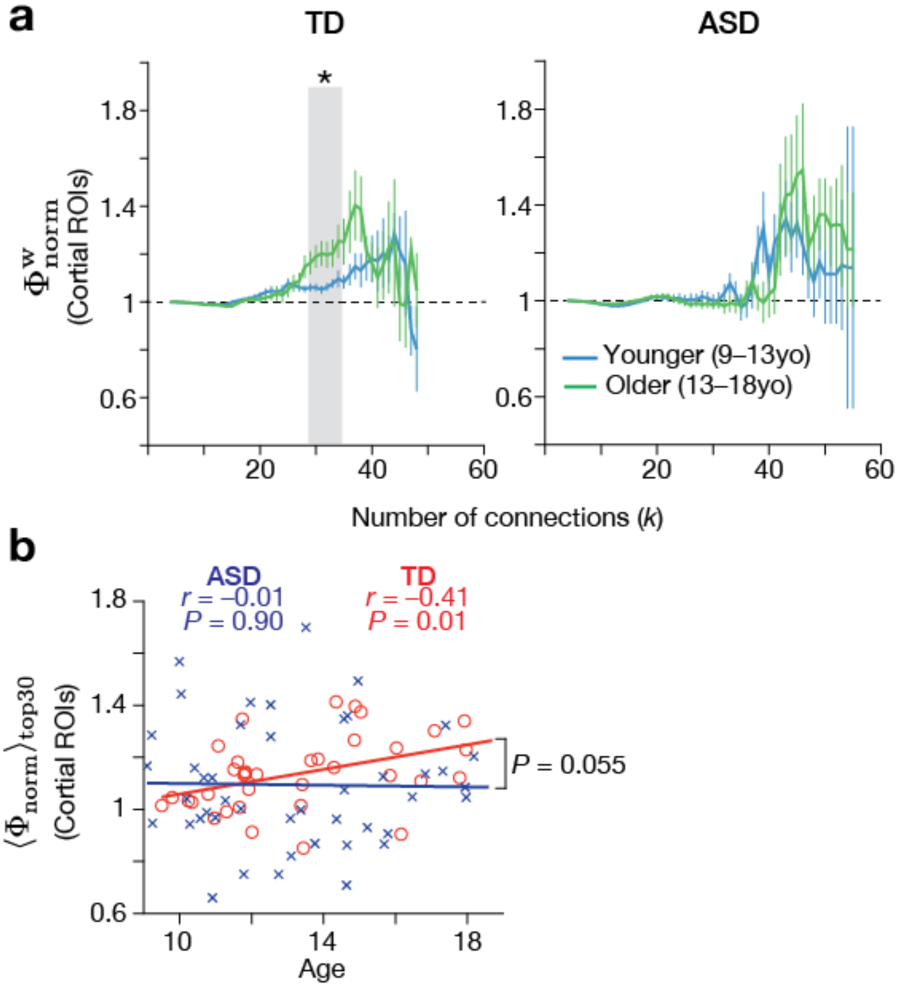

Results based on cortical networks.

To evaluate influence of the subcortical regions on the current findings, we estimated rich-club coefficients using brain networks consisting of 244 cortical ROIs. **(a)** As a result, we observed the similar pattern in the comparison of the coefficients between TD/ASD and younger/older groups. **(b)** An age-related change was also seen in TD group, but the difference in the age-related changes between TD and ASD groups were slightly milder than that observed in the original whole-brain networks consisting of 264 ROIs including the subcortical areas such as the thalamus. *, $P_{FDR} < 0.05$ in $k = 29$–34.

Supplementary Results

Effects of $\langle k \rangle$ and $\langle W \rangle$ on $\langle \Phi^{\mathrm{w}}_{\mathrm{norm}} \rangle_{\mathrm{top30}}$:

Age-related neuroanatomical changes could affect the detectability of fibres in DTI. In fact, in the current data, age of TD individuals was significantly correlated with both the average degree, $\langle k \rangle$, and weight, $\langle W \rangle$ (age v $\langle k \rangle$: $r = 0.35$, $P = 0.035$; age v $\langle W \rangle$: $r = 0.43$, $P = 0.0091$). Therefor, we attempted to reduce this confounding effect by dividing $\langle \Phi^{\mathrm{w}}_{\mathrm{norm}} \rangle_{\mathrm{top30}}$ by $\langle k \rangle$ or $\langle W \rangle$. Such correction was supposed to normalise the difference in $\langle k \rangle$ or $\langle W \rangle$ between TD and ASD groups, though originally there was no significant difference.

Supplementary Methods

Analyses based on binary brain networks.

A binary network matrix, A, was built for each participant by defining a connection, A_{ij}, between regions i and j as follows: $A_{ij} = 1$, when $W_{ij} > 0$; otherwise, $A_{ij} = 0$, where W_{ij} represents the number of the fibres detected between region i and j. Using these binary matrices, we calculated normalised rich-club coefficients in the same manner as in previous studies[3-5]: we first estimated an un-normalised rich-club coefficient, $\Phi(k)$, for each degree as follows:

$$\Phi(k) = \frac{2E_{>k}}{N_{>k}(N_{>k} - 1)},$$

where $E_{>k}$ indicates the number of connections among $N_{>k}$ regions that have more than k degrees. Before normalisation, we designed 5000 different random networks with the same degree distribution as the original one with the method adopted in previous studies[2,6]. We then estimated rich-club coefficients for the 5000 random networks and estimated the averaged coefficient, $\Phi_{\mathrm{random}}(k)$. The normalised rich-club coefficient, $\Phi_{\mathrm{norm}}(k)$, was then calculated as

$$\Phi_{\mathrm{norm}}(k) = \frac{\Phi(k)}{\Phi_{\mathrm{random}}(k)}.$$

We used these unweighted rich-club coefficients to investigate relationship between age and rich-club property.

Global efficiency for the binary networks was estimated in essentially the same manner as one adopted for weighted connectivity matrices: we first estimated the shortest path length among all region pairs[7,8], and calculated the global efficiency as

$$\frac{\sum_{i \neq j} \frac{1}{\ell_{ij}^{\mathrm{binary}}}}{N(N-1)},$$

where N represents the number of the regions and $\ell_{ij}^{\mathrm{binary}}$ denotes the shortest path length between region i and j.

Synchronisation cost for a binary matrix is also defined in essentially the same manner as that for a weighted matrix. A complex network consisting of Kuramoto-type phase oscillators with different natural frequencies was first defined by the following equation:

$$\frac{d\theta_i}{dt} = \omega_i - \epsilon \sum_j A_{ij} \sin(\theta_i - \theta_j),$$

where θ_i and ω_i indicate the phase and natural frequency of the oscillator *i.* The coupling strength, ϵ, was set as a constant value, 0.3 (ref[9]). The synchronisation cost, S_{ij}^{binary}, for an edge between nodes *i* and *j* was defined as $S_{ij}^{\text{binary}} = (\theta_i^{t\to\infty} - \theta_j^{t\to\infty})^2$, where $\theta_i^{t\to\infty}$ and $\theta_j^{t\to\infty}$ indicate the phases of the synchronised oscillators *i* and *j*, respectively. We finally calculated the average synchronisation costs across edges for each individual.

Estimation of group-averaged rich-club coefficients

We estimate group-average rich-club coefficients in the same manner as in a previous study[2]. First, we built a binary connectivity matrix, *A*. Second, a group-average connectivity matrix, $\bar{A}$, was built for each of the four groups (i.e., younger/older × TD/ASD groups) by selecting region pairs that were connected in at least 75% of the participants in each group. Third, group-averaged weights were calculated only for the connected pairs of regions (i.e., $\bar{A}_{ij} = 1$). If two different regions were not averagely connected (i.e., $\bar{A}_{ij} = 0$), the corresponding group-averaged weight was defined as 0. For each of these four group-averaged weighted connectivity matrices, we finally calculated rich-club coefficients in the same manner as described in the main text (see "Estimation of rich-club coefficients" in Methods section).

Supplementary References

1. Ray, S. *et al.* Structural and functional connectivity of the human brain in autism spectrum disorders and attention-deficit/hyperactivity disorder: A rich club-organization study. *Hum Brain Mapp* **35,** 6032–6048 (2014).
2. van den Heuvel, M. P. & Sporns, O. Rich-club organization of the human connectome. *J. Neurosci.* **31,** 15775–15786 (2011).
3. Zhou, S. & Mondragon, R. J. The rich-club phenomenon in the Internet topology. *Communications Letters, IEEE* **8,** 180–182 (2004).
4. Colizza, V., Flammini, A., Serrano, M. A. & Vespignani, A. Detecting rich-club ordering in complex networks. *Nat Phys* **2,** 110–115 (2006).
5. Gómez-Gardeñes, J., Zamora-López, G., Moreno, Y. & Arenas, A. From modular to centralized organization of synchronization in functional areas of the cat cerebral cortex. *PLoS ONE* **5,** e12313 (2010).
6. Maslov, S. & Sneppen, K. Specificity and stability in topology of protein networks. *Science* **296,** 910–913 (2002).
7. Dijkstra, E. W. A note on two problems in connexion with graphs. *Numerische Mathmatik* **1,** 269–271 (1959).
8. Newman, M. E. Scientific collaboration networks. II. Shortest paths, weighted networks, and centrality. *Phys. Rev. E* **64,** 016132 (2001).
9. Watanabe, T. Rich-club network topology to minimize synchronization cost due to phase difference among frequency-synchronized oscillators. *Phys. A* **392,** 1246–1255 (2013).
